# Identification of disulfidptosis-related genes and immune infiltration in lower-grade glioma

**DOI:** 10.1515/med-2023-0825

**Published:** 2023-10-25

**Authors:** Xiao-min Li, Shan-peng Liu, Dan-man Liu, Yu Li, Xiao-ming Cai, Yun Su, Ze-feng Xie

**Affiliations:** The First Affiliated Hospital of Shantou University Medical College, Shantou, Guangdong, China; Laboratory of Brain Disorders, Ministry of Science and Technology, Joint Innovation Center for Brain Disorders, Beijing Institute of Brain Disorders, Capital Medical University, Beijing, China; Department of Microbiology & Immunology, Shantou University Medical College, 22 Xinling Road, Shantou 515041, Guangdong, China; Breast Surgery Clinics, Guangdong Province Women and Children Hospital, Guangzhou, China

**Keywords:** disulfidptosis, lower-grade glioma, immune infiltration, machine learning, MCL1

## Abstract

Lower-grade glioma (LGG), a prevalent malignant tumor in the central nervous system, requires accurate prediction and treatment to prevent aggressive progression. We aimed to explore the role of disulfidptosis-related genes (DRGs) in LGG, a recently discovered form of programmed cell death characterized by abnormal disulfide accumulation. Leveraging public databases, we analyzed 532 LGG tumor tissues (The Cancer Genome Atlas), 1,157 normal samples (Genotype-Tissue Expression), and 21 LGG tumor samples with 8 paired normal samples (GSE16011). Our research uncovered intricate relationships between DRGs and crucial aspects of LGG, including gene expression, immune response, mutation, drug sensitivity, and functional enrichment. Notably, we identified significant heterogeneity among disulfidptosis sub-clusters and elucidated specific differential gene expression in LGG, with myeloid cell leukemia-1 (MCL1) as a key candidate. Machine learning techniques validated the relevance of MCL1, considering its expression patterns, prognostic value, diagnostic potential, and impact on immune infiltration. Our study offers opportunities and challenges to unravel potential mechanisms underlying LGG prognosis, paving the way for personalized cancer care and innovative immunotherapeutic strategies. By shedding light on DRGs, particularly MCL1, we enhance understanding and management of LGG.

## 1 Introduction

Gliomas are the most common primary intracranial malignant tumors of the central system, with approximately 50% of patients exhibiting aggressiveness [1]. Gliomas are mainly classified as lower-grade glioma (LGG) and glioblastoma multiforme (GBM). LGG is considered a worldwide health problem, accounting for approximately 20% of gliomas diagnosed in the United States [2]. Although LGG is less aggressive than GBM, there is a higher incidence in young people and a tendency to progress to high grade in later stages [3]. Therefore, efficient and sensitive diagnostic novel markers are needed to predict prognosis and stop progression.

Research is now proposing a novel mode of cell death, disulfidptosis, independent of the currently existing programmed cell death such as apoptosis, ferroptosis, necroptosis, and cuproptosis. It is a rapid form of death caused by disulfide stress resulting from the accumulation of excess intracellular cystine [4]. Earlier studies found that under glucose starvation conditions, NADPH was heavily depleted in SLC7A11 overexpressing cells and disulfides such as cystine accumulated abnormally, inducing disulfide stress and rapid cell death [5]. The endoplasmic reticulum of eukaryotic cells and the periplasmic space of prokaryotic cells are capable of forming and transferring protein disulfide bonds. The formation of structural disulfide bonds is a catalytic process involving many proteins and small molecules [6]. The formation of disulfide bonds has now been identified in cancer-related proteins and it is time to consider how this allosteric bond can be used as a target for new therapies [7]. In addition, a variety of disulfide isomerases have been shown to be associated with tumorigenicity in a variety of tumors [8,9]. Notably, protein disulfide isomerase may play a role in the malignant progression of gliomas and predict the clinical prognostic value of gliomas [10]. Furthermore, RAC1, a component of the WAVE regulatory complex, plays a significant role in actin polymerization and the formation of thin-walled cells, processes that are implicated in disulfidptosis. The RAC1 signaling pathway has been linked to epileptogenic mechanisms in glioma-associated epilepsy [11]. However, the precise impact of disulfidptosis on the prognosis and immune infiltration of LGG is still unclear and requires further investigation.

To explore possible pathogenic mechanisms, we analyzed genes differentially expressed between LGG samples and normal tissue using Cancer Genome Atlas (TCGA) and Gene Expression Omnibus (GEO) databases. Disulfidptosis-related gene (DRGs) were interrogated and explored for expression, immunity, mutation, and drug sensitivity in LGG. Sub-clusters of DRGs were then constructed based on clinical features and gene expression and the associated mechanisms explored. Differential genes and DRGs were then extracted for crossover to find the differentially expressed DRGs. In addition, machine learning algorithms were applied to find key differential genes. Finally, the strongest trait gene was identified and the relationship with prognosis and immune infiltration was further considered in LGG. This provides a new perspective to better understand the underlying molecular mechanisms of LGG pathogenesis.

## 2 Materials and methods

### 2.1 Identification of DRGs

We identified nine DRGs from the previous literature [12]. This study identified and selected DRG genes based on their consistent and notable association with disulfidptosis, a cellular process of interest. Each gene encodes specific proteins that play crucial roles in biochemical pathways and cellular structures involved in disulfidptosis. Among these genes, SLC7A11 was emphasized due to its central role as a cystine transporter in this process. Additionally, the selection criteria included genes that interact with the WAVE regulatory complex (NCKAP1, WASF2, CYFIP1, ABI2, BRK1, and RAC1), which is known to be involved in actin polymerization and the formation of lamellipodia, both relevant to disulfidptosis. Moreover, BAK1 and ACSL4 were chosen for their significant roles in regulating programmed cell death and their close association with disulfidptosis. GeneMANIA Prediction Server is a biological network integration for gene prioritization and prediction of gene function [13]. We used the GeneMANIA website (http://www.genemania.org) to identify functionally similar genes and create 29 DRGs.

### 2.2 LGG datasets

LGGs are a group of primary brain tumors that originate from glial cells. Currently, LGGs include WHO grade II and III gliomas, and their classification is based on molecular features rather than histopathological characteristics [14]. The RNA-sequencing (RNA-seq) data and relevant clinical data of 532 LGG tumor tissues were downloaded from TCGA (http://cancergenome.nih.gov) and Genotype-Tissue Expression (GTEx) database (https://www.gtexportal.org/home/-index.html) of LGG normal was extracted (*n* = 1,157). The above datasets were selected to meet the criteria at the time of the last data freeze in spring 2023. In addition, the GSE16011 dataset [15] was derived from GEO (https://www.ncbi.nlm.nih.gov/geo/.), which contains 21 LGG cancer samples and 8 paired normal samples. Two datasets are used to explore DRGs’ expression levels, differential gene analysis, machine learning, prognosis, and immune infiltration, among others. Data were extracted in TPM format and further log2(*x* + 1) transformations were performed for each expression value. All data analyses was carried out using R (version 4.2.1) and the relevant bioinformatics analysis website.

### 2.3 Gene set and differentially expressed gene (DEG) functional enrichment analysis

For gene set functional enrichment, we used the kyoto encyclopedia of genes and genomes (KEGG) rest API (https://www.kegg.jp/kegg/rest/keggapi.html) to obtain the latest KEGG pathway gene annotations as background, mapped the genes to the background set, and used the R package ClusterProfiler (version 3.14.3) [16] to perform the enrichment analysis to obtain the results of gene set enrichment. Similarly, we used the Gene Ontology (GO) annotations of genes from the R package org.Hs.eg.db (version 3.1.0) as a background, mapped the genes to the background set, and used the R package ClusterProfiler (version 3.14.3) to perform enrichment analysis to obtain the gene set enrichment results. *p* values < 0.05 and false discovery rate (FDR) < 0.1 were considered statistically significant.

For DEGs’ functional enrichment, KEGG enrichment analysis is practical for analyzing gene function and associated high-level genomic functional information. GO is a widely used tool for the annotation of genes with functions, in particular molecular function, biological pathway, and cellular component. To better understand the oncogenic and immune infiltration of target genes, we obtained enrichment results for differentially up/down-regulated genes KEGG pathway and enrichment results for differentially up/down-regulated genes GO term. The functional enrichment results were obtained from the R package ClusterProfiler (version:3.18.0). *p* values <0.05 were considered statistically significant.

### 2.4 Gene set cancer analysis (GSCA)

GSCA is an integrated platform for genomic, pharmacogenomic, and immunogenomic cancer analysis [17]. Within this enhanced GSCA, a range of services are provided to perform gene set genomic including expression, single-nucleotide variation (SNV), copy number variation (CNV), methylation, and immunogenomic (24 immune cells) analysis. In addition, the combination of clinical information and small molecule drugs allows the mining of candidate biomarkers and valuable small drugs to inform further clinical trials.

### 2.5 Subgroup analysis

Consistency analysis was performed using the ConsensusClusterPlus R package (v1.54 4.0) [18] with a maximum number of clusters of 6 and 80% of the total sample drawn 100 times, clusterAlg = “hc,” innerLinkage = “ward.D2.” Cluster heatmaps were performed using the R package pheatmap (v1.0.12). Gene expression heatmaps retained genes with an SD of >0.1.

### 2.6 Differential genetic screening

Limma (linear models for microarray data) is a differential expression screening method that utilizes a generalized linear model [19]. In the TCGA database, DEGs were screened in subgroup C1 and C2 data sets. Differentially expressed mRNA was studied using the limma package (version 3.40.6). Threshold for differential mRNA expression between two clusters was set at “Adjusted *p* < 0.05 and |log2 FC| > 1.” In GSE16011, *p* < 0.05 and |log2 FC| > 1.5 was selected as the cut-off standard.

### 2.7 Machine learning

To identify trait genes, two machine learnings were used to screen for DRGs. The least absolute shrinkage and selection operator (LASSO) is a regression method used for regularization to improve prediction accuracy and model comprehensibility by select variables [20]. Random Forest is a learning method that constructs a large number of decision trees and outputs classes of individual trees. This method has a high degree of accuracy, sensitivity, and specificity [21]. Log rank was used to test survival analysis comparing survival differences between two groups, and timeROC analysis was performed to discriminate the accuracy of the predictive model.

### 2.8 Statistical analysis

All statistical tests were performed using the R package (version 4.2.1) and visualizations were performed using the ggplot2 package (version 3.3.6). Expression correlation network of the DRGs analysis and visualization using igraph package (version 1.3.4) and ggraph package (version 2.1.0). With the xCell package (version 1.1.0), the integrated level of 64 cell types was estimated, including 14 stromal cell types. LASSO regression and Random Forest analyses were carried out using the R packages “glmnet” [22] and “randomForest” [23]. Kaplan–Meier survival analyses were performed with the “survival R” and “survminer R” packages (version 3.3.1). ROC analysis was performed with the qROC package (version 1.18.0). Construction and visualization of Nomogram models were carried out using the rms package (version 6.4.0). Spearman correlation analysis was used to understand the relationship between myeloid cell leukemia-1 (MCL1) expression levels and immune infiltration. The immune infiltration algorithm (ssGSVA) in the GSVA package (version 1.46.0) was used to calculate immune scores [24]. Wilcoxon rank-sum test was used to compare differences between groups. *p* < 0.05 was considered to indicate statistical significance (ns, *p* ≥ 0.05; ∗*p* < 0.05; ∗∗*p* < 0.01; ∗∗∗*p* < 0.001).

## 3 Results

### 3.1 Assessing differential expression of DRGs in LGG

**Figure 1:**
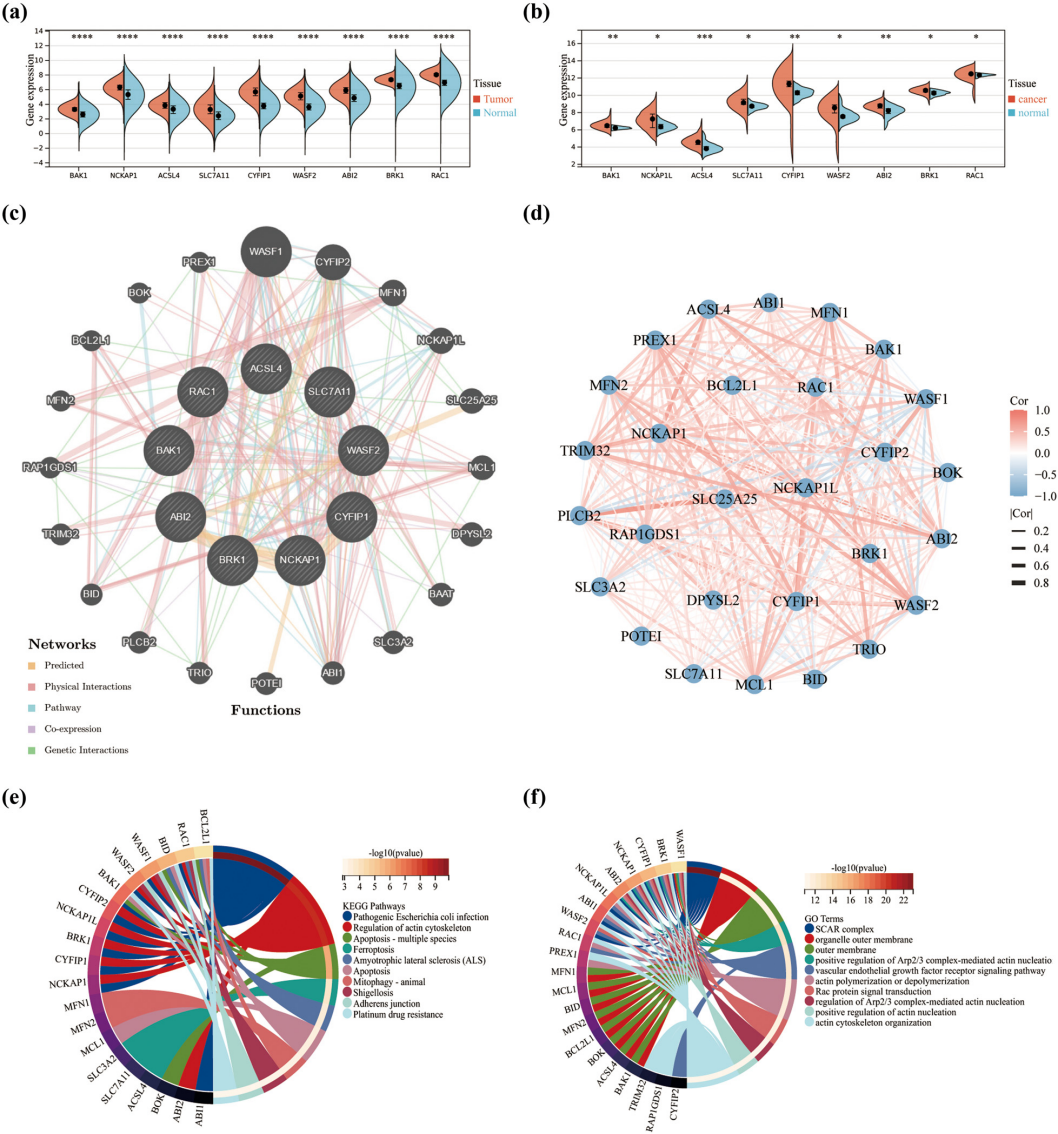
Expression distributions, Spearman correlation, and enrichment analysis of DRGs in LGG. Expression distributions of nine DRGs between cancer and normal tissues in the (a) TCGA and (b) GEO datasets. (c) GeneMANIA website for identifying functionally similar genes and establishing 29 DRGs. Twenty similar genes are located in the outer circle, while nine hub genes are located in the inner circle. Five colors of the lines represent the type of gene interactions. (d) Expression correlation network of the DRGs. Positive correlations are shown by the red line and negative correlations are shown by the blue line. The thickness of the line indicates the strength of the correlation. (e) KEGG and (f) GO concentrated circle diagram. *p < 0.05, **p < 0.01, ***p < 0.001 and ****p < 0.0001.

As previously described, nine genes (BAK1, NCKAP1, ACSL4, SLC7A11, CYFIP1, WASF2, ABI2, BRK1, and RAC1) were shown to be associated with disulfidptosis [12]. To confirm the expression of these related genes in LGG, we downloaded expression data from the TCGA and GTEx databases for cancer and normal tissues, which showed differences in the expression of DRGs. All relevant genes were upregulated and significant in tumor expression. Consistent results were also obtained in the GEO database (Figure 1(a) and (b)). We used GeneMANIA on predicting functionally similar genes in hub genes. We obtained 20 similar gene hub genes, comprising WASF1, CYFIP2, MFN1, NCKAP1L, SLC25A25, MCL1, DPYSL2, BAAT, SLC3A2, ABI1, POTEI, TRIO, PLCB2, BID, TRIM32, RAP1GDS1, MFN2, BCL2L1, BOK, and PREX1. The hub gene was located in the inner circle, while the predicted genes were in the outer circle. The relationships between genes were specifically based on five types, including Predicted, Physical Interactions, Pathway Co-expression, and Genetic Interactions (Figure 1(c)). Combined with the network diagram of correlation analysis, most of the related gene expressions were positively correlated with each other (Figure 1(d)). Enrichment analysis of DRGs in the KEGG dataset identified some apoptosis and disease-related pathways such as pathogenic *Escherichia coli* infection, regulation of actin cytoskeleton, apoptosis-multiple species, ferroptosis, amyotrophic lateral sclerosis, apoptosis, and so on (Figure 1(e)). Further enrichment analysis of these genes on the GO dataset indicated that certain related actin nucleation items, such as organelle outer membrane, outer membrane, positive regulation of Arp2/3 complex-mediated actin nucleation, vascular endothelial growth factor (VEGF) receptor signaling pathway, actin polymerization or depolymerization, regulation of Arp2/3 complex-mediated actin nucleation, positive regulation of actin nucleation, actin cytoskeleton organization, and so on (Figure 1(f)). The above analysis shows that DRGs are confirmed with some reliability in LGG.

### 3.2 Exploring the expression, immunity, mutations, and drug sensitivity of 29 DRGs in LGG

**Figure 2:**
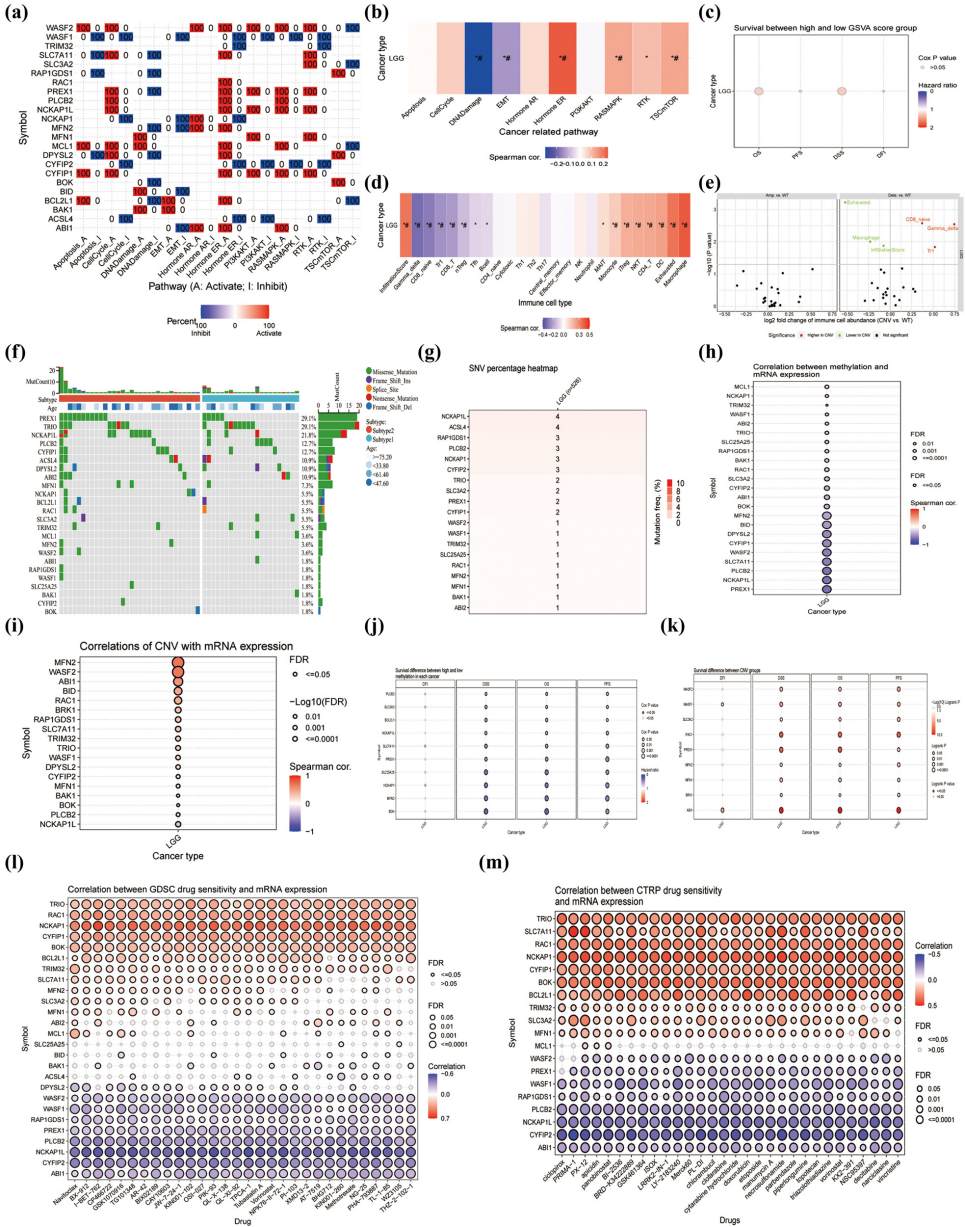
Expression, immunity, mutations, and drug sensitivity of 29 DRGs in LGG. (a) Percentage of LGG in which mRNA expression of specific genes has a potential effect on pathway activity. (b) The association between GSVA score and activity of cancer-related pathways in LGG. (c) The results of survival difference between GSVA score groups in LGG. (d) The association between GSVA score and activity of cancer-related pathways in LGG. (e) the difference of immune infiltration between gene set CNV groups. (f) Waterfall plot showing the mutational landscape of DRGs in LGG. (g) The profile of SNV of the DRGs set in LGG. (h) The profile of correlations between methylation and mRNA expression of DRGs in LGG. (i) The correlations between CNV and mRNA expression in LGG. (j) The OS difference between higher and lower methylation groups in LGG. (k) The difference of survival between CNV and wide type in LGG. Correlation of gene expression with (l) GDSC drug sensitivity (top 30) and (m) CTRP drug sensitivity (top 30) in pan-cancer. DFI: disease-free interval; DSS: disease-specific survival; OS: overall survival; PFS: progression-free survival; GSVA score: gene set expression score; CNV: copy number variation; SNV: single-nucleotide variation; Amp: amplification; Dele: deletion; WT: wild type; CTRP: The Cancer Therapeutics Response Portal. *p < 0.05, **#**FDR <0.05.

To gain a comprehensive understanding of the role and relevance of DRGs in cancer diagnosis, we used GSCA to further correlate the analysis of four modules, including expression, immunity, mutations, and drug sensitivity. In the expression module, summarize the percentage of LGG for which specific gene mRNA expression has a potential impact on pathway activity (Figure 2(a)). Specific pathways include: Apoptosis, Cellcycle, DNA damage, EMT, Hormone AR, Hormone ER, PI3KAKT, RASMAPK, RTK, and TSCmTOR. Specifically, gene sets were most meaningfully and positively correlated with Hormone ER (*p* < 0.05, #FDR < 0.05) and most negatively correlated with DNA Damage pathways (*p* < 0.05, #FDR < 0.05) (Figure 2(b)). Moreover, the gene set has an impact on patient survival outcomes, with overall survival (OS) and disease-specific survival close but less significant (*p* > 0.05) (Figure 2(c)). In the immunity module, gene sets were most meaningfully and positively correlated with macrophage (*p* < 0.05, #FDR < 0.05) and most negatively correlated with Gamma delta (*p* < 0.05, #FDR < 0.05) (Figure 2(d)). Interestingly, Figure 2(e) summarizes the difference of immune infiltration between gene set CNV groups. CD8 native, Gamma delta, and Tr1 are meaningfully highly expressed in CNV, as opposed to Exhausted, Macrophage, and InfiltrationScore. In the mutation module, we can see that the waterfall plot is dominated by Missense mutation, with PREX1 and TRIO reaching the highest 29.1% (Figure 2(f)). NCKAP1L and ACSL4 were the most frequent mutants (Figure 2(g)). CNV induced the extensive upregulation of its mRNA expression (Figure 2(i)) and survival (Figure 2(k)). Methylation, however, affected extensive downregulation of their mRNA expression (Figure 2(h)) and survival (Figure 2(j)). Furthermore, the correlation of gene expression with the genomics of drug sensitivity in cancer (GDSC) drug sensitivity (top 30) (Figure 2(l)) and Cancer Therapeutics Response Portal (CTRP) drug sensitivity (top 30) (Figure 2(m)) in pan-cancer demonstrates consistency and reliability of results. In conclusion, analysis of the multiple modules described above showed strong associations in terms of expression, immune infiltration, mutations, and drug sensitivity of DRGs in LGG.

### 3.3 Two disulfidptosis sub-clusters and analysis of associated differential genes

**Figure 3:**
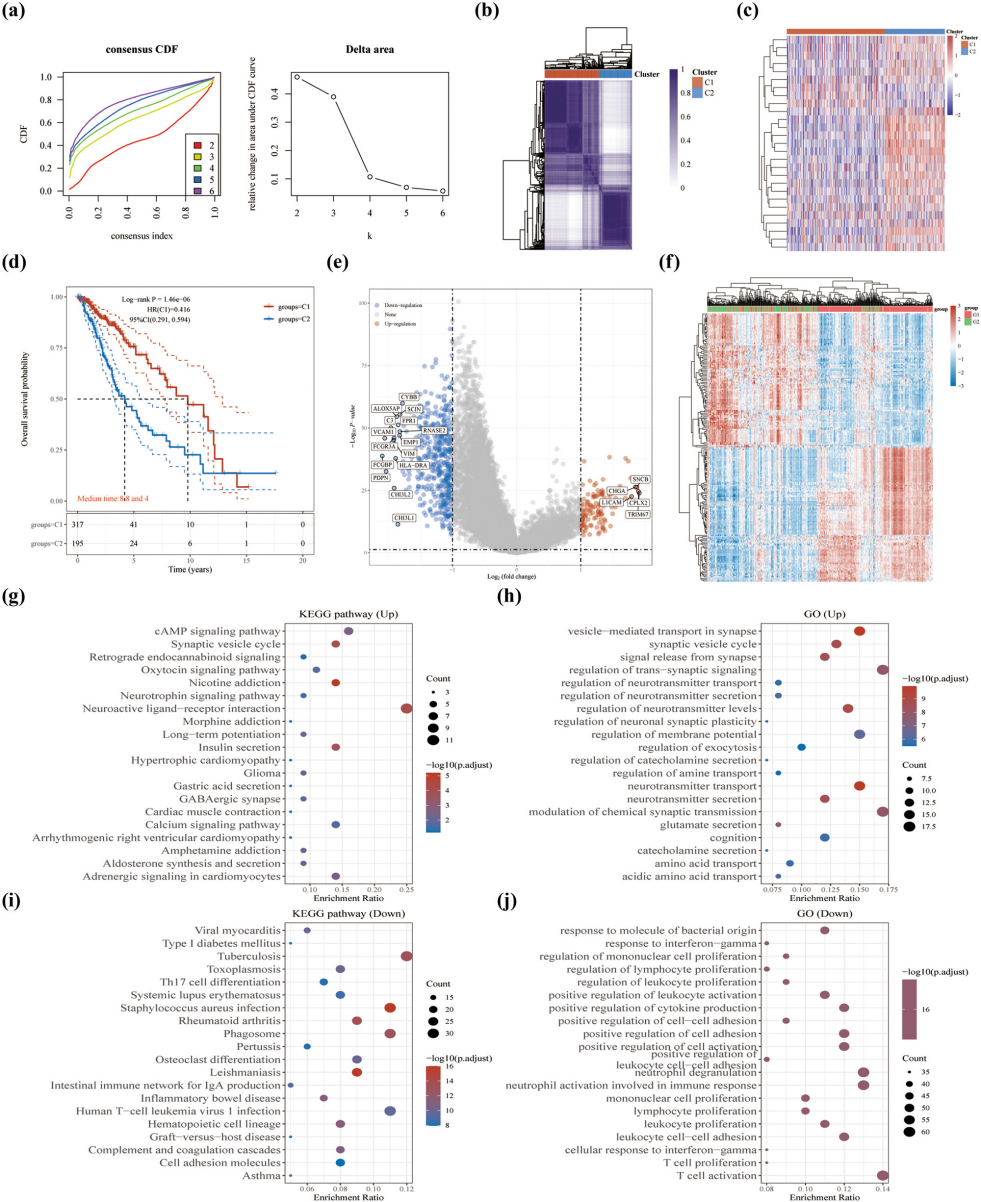
Two disulfidptosis sub-clusters were shown. (a) Cumulative distribution function (CDF) curve of K (2–6). The relative change in area under the CDF curve of K (2–6). (b) Appropriate unsupervised clustering analysis (*k* = 2). (c) Heat map showing the relationship between DRGs’ expression in subgroups. (d) Survival curve analysis revealed differences in OS between 2 subgroups. Two groups were tested by log rank, with 95% CL representing the HR confidence interval; median time represents the time in years corresponding to survival in the different groups at 50%. (e) Differential analysis of subgroups. (f) Heatmap showing DEGs. (g–j) KEGG pathway and GO air bubble diagram. In the enrichment result, *p* values <0.05 are considered to be a meaningful pathway (enrichment score with −log10 (P) of more than 1.3).

**Figure 4:**
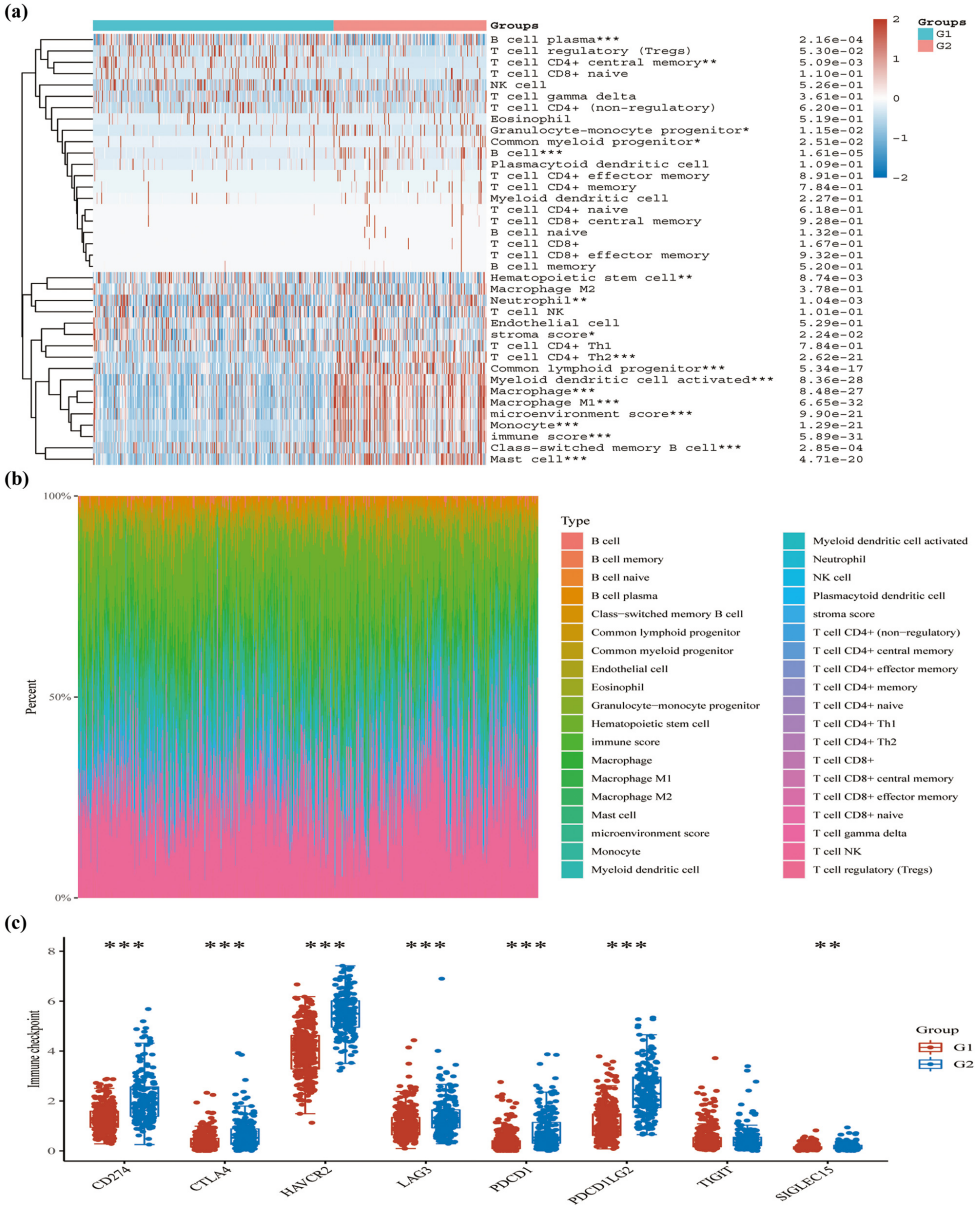
Immune cells’ infiltration between subgroups. (a) Immune cells’ infiltration between different groups by xCell algorithms. (b) The proportion structure of all Immune cell types. (c) The expression of eight key immune checkpoint genes in two subgroups. *p < 0.05, **p < 0.01 and ***p < 0.001.

We performed consensus unsupervised clustering on a sample of 512 patients in LGG from TCGA databases, with 2 clusters (Cluster 1 [*n* = 317], Cluster 2 [*n* = 195]) selected for relative stability under the distribution (Figure 3(a) and (b)). In contrast to the C1 subgroup, DRGs were highly expressed in the C2 subgroup (Figure 3(c)) and had a poorer prognosis on the survival curve (Figure 3(d)). To further explore the differences between the two subgroups, Limma analysis of the volcano map (Figure 3(e)) and heat map (Figure 3(f)) was used to demonstrate the differential genes between the two subgroups. 115 upregulated genes such as SNCB, CHGA, LICAM, CPLX2, TRIM67 and 473 downregulated genes were identified in DRGs-high group such as CYBB, SCIN, C3, FPR1, ALOX5AP were identified in the DRGs-low group (C1) compared to DRGs-high group (C2) (Figure 3(e)). Enrichment analysis of the upregulated genes in the KEGG dataset is shown in the neuroactive ligand−receptor interaction pathway (Figure 3(g)), compared to the downregulated genes in the tuberculosis, *Staphylococcus aureus* infection, and phagosome pathways (Figure 3(i)). The further enrichment analysis of the upregulated genes in the GO dataset is shown in the regulation of trans-synaptic signaling, modulation of chemical synaptic transmission, and vesicle-mediated transport in synapse (Figure 3(h)), compared to the downregulated genes in T-cell activation, neutrophil activation involved in immune response, and neutrophil degranulation, which were involved in immune response (Figure 3(j)). In addition, the xCell algorithms were used to analyze the immunological characteristics of subgroups (Figure 4(a)). Compared to C1 subgroup, C2 subgroup showed a meaningful positive correlation with immune cells. The proportions of all immune cell types are shown in Figure 4(b). To predict the effect of immune checkpoint blockade therapy, we also explored the expression of key immune checkpoint genes in the groups (Figure 4(c)). The results showed that the expressions of CD274, CTLA4, HAVCR2, LAG3, PDCD1, PDCD1LG2, and SIGLEC15 were elevated in C2 subgroup (Figure 4(c)), which suggested an immunosuppressive status.

### 3.4 Construction of prognosis risk model based on DRGs in TCGA dataset

**Figure 5:**
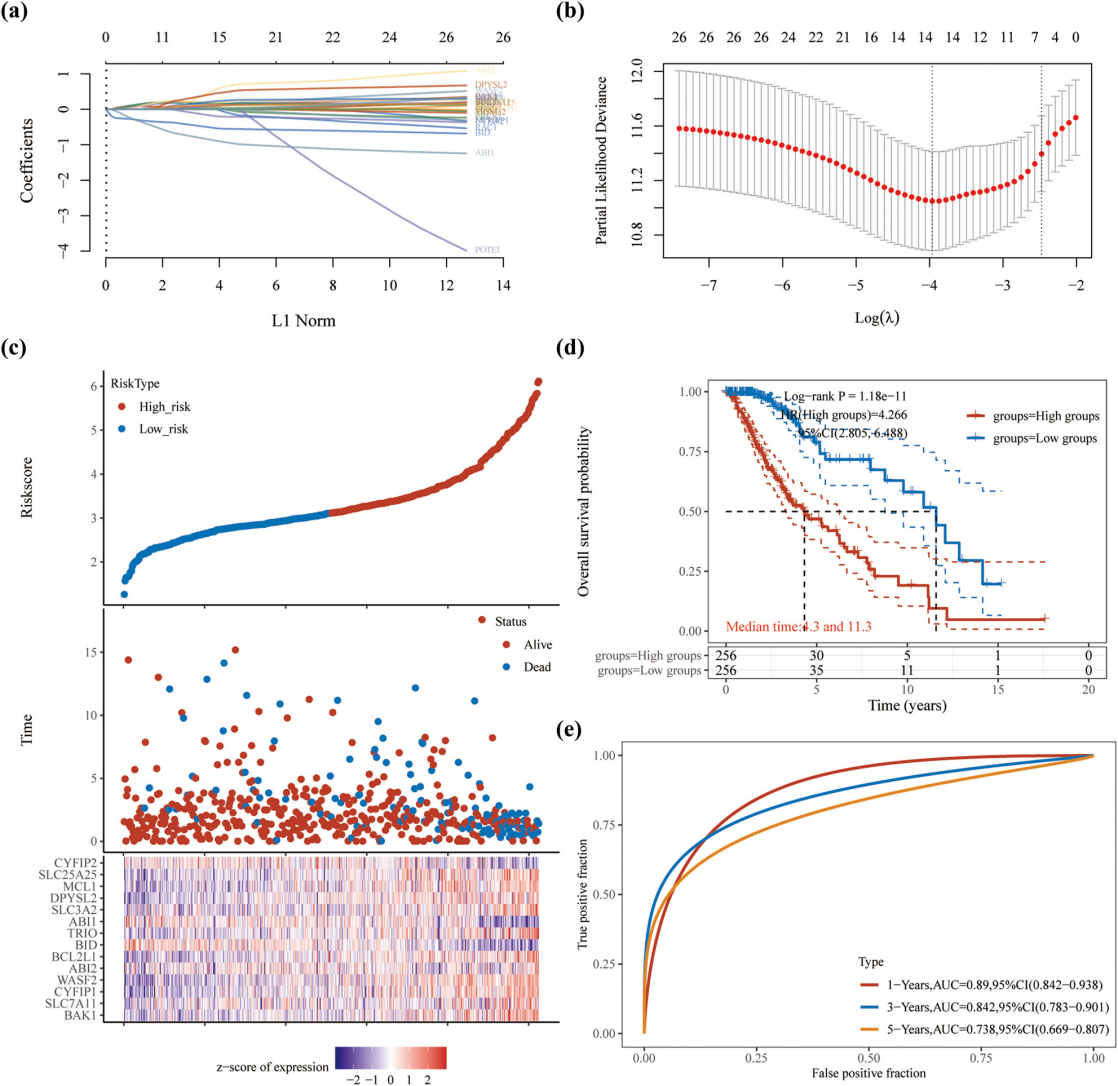
Evaluation of disulfidptosis signature by the performance of the 14-gene signature in the TCGA dataset. (a) Construction of disulfidptosis signatures using LASSO regression. (b) Determining the appropriate number of genes by confidence intervals of lambda. (c) Risk score, survival time, and expression of the 14-gene signature in LGG. (d) Kaplan–Meier survival analysis of OS was compared between low- and high-risk score groups in LGG. (e) ROC curves over time at 1, 3, and 5 years, respectively.

To identify new prognostic markers for LGG, we performed a LASSO regression analysis of LGG patients in the TCGA database based on 29 DRGs. The LASSO regression algorithm used 10-fold cross-validation for feature selection, and all genes except POTEI showed consistency (Figure 5(a)). Finally, 14 genes were identified with disulfidptosis-signature, including BAK1, SLC7A11, CYFIP1, WASF2, ABI2, BCL2L1, BID, TRIO, ABI1, SLC3A2, DPYSL2, MCL1, SLC25A25, and CYFIP2 (Figure 4(b) and (c)). We also confirmed that OS was significantly longer in the low-risk group than in the high-risk group (hazard ratio [HR] = 4.266, 95% confidence interval [CI] = 1.78–2.89, *P* < 0.001), comparing median times of 11.3–4.3 years, respectively (Figure 5(d)). Meanwhile, ROC time-dependent curves demonstrated that the accuracy of 14 gene signatures was greater than 0.70 for 1-, 3-, and 5-year survival rates (area under curve > 0.7 indicates a high degree of accuracy) (Figure 5(e)).

### 3.5 Application of machine learning to the identification of trait genes via the GEO dataset

**Figure 6:**
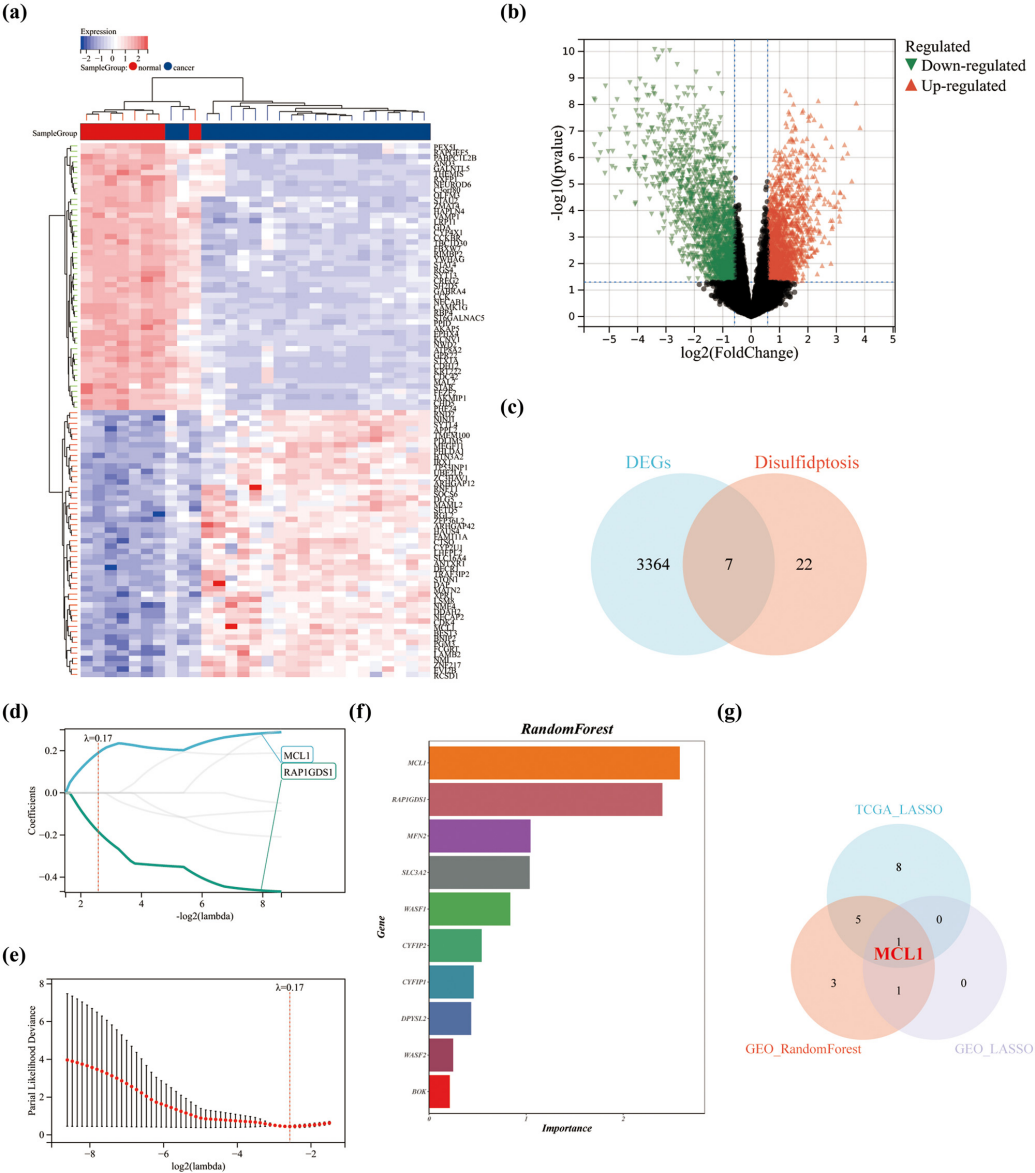
Machine learning to identify trait genes in the GEO dataset. Differential genes in GSE16011 showing (a) heat map and (b) volcano map. (c) Trait genes shared by DRGs and differential genes of GSE16011. (d and e) Selection of the most relevant trait genes using LASSO regression. (f) Selection of the most relevant DRGs based on GSE16011 using RandomForest (top 10). (g) The Venn diagram shows the overlap of candidate genes between the two databases.

To verify the reliability of the above analysis based on TCGA data, we also performed further machine learning analysis of LGG patients based on the GEO database. First, we used Limma analysis of heat map (showed top 50 up/downregulated genes respectively) (Figure 6(a)) and volcano map (Figure 6(b)) to demonstrate differential genes in LGG patients in GSE16011. A total of 1,533 upregulated genes and 1,818 downregulated genes were identified, 7 of which were associated with DRGs (Figure 6(c)). Second, LASSO regression was used to select the most relevant trait genes. When *λ* = 0.17, MCL1 and RAP1GDS1 were selected (Figure 6(d) and (e)). Meanwhile, we used RandomForest algorithm to screen DRGs and construct potential genes based on the GSE16011 dataset. We show the top 10 genes in order, including MCL1, RAP1GDS1, MFN2, SLC3A2, WASF1, CYFIP2, CYFIP1, DPYSL2, WASF2, and BOK (Figure 6(f)). Ultimately, MCL1 was selected as the only candidate gene (Figure 6(g)).

### 3.6 Clinical diagnosis and prognostic value analysis of MCL1-related gene marker

**Figure 7:**
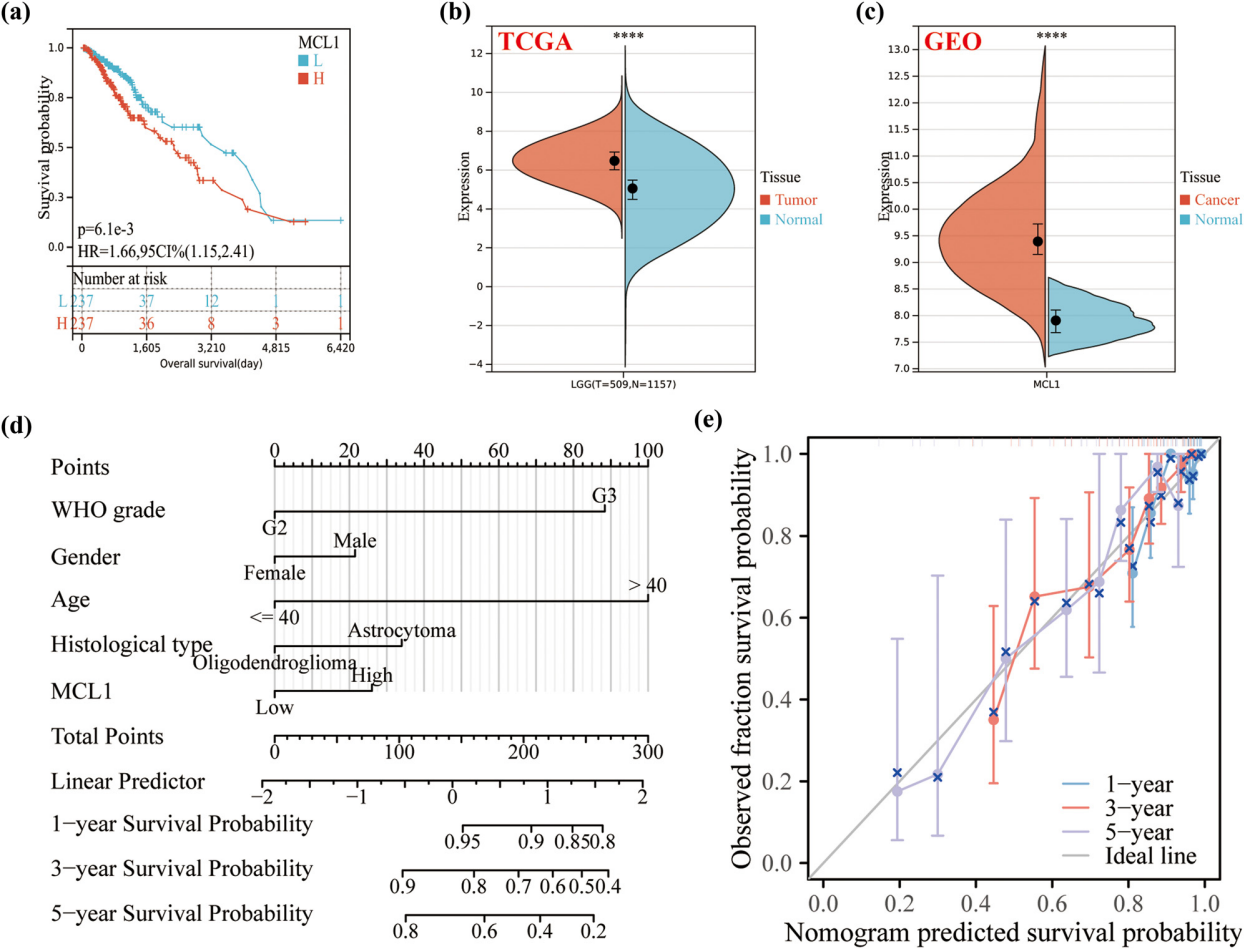
Construction of nomogram for OS prediction based on TCGA. (a) Kaplan–Meier survival analysis of LGG patients in the high-risk and low-risk groups. Expression distributions of MLC1 between cancer and normal tissues in the (b) TCGA and (c) GEO datasets. (d) A nomogram combining MLC1 and clinicopathological features from TCGA LGG data. (e) Nomogram calibration curve for predicting OS in TCGA LGG data.

In the present study, we found that MCL1 expression was upregulated in LGG patients compared to normal tissue by analyzing data from the TCGA and GTEx databases (Figure 7(b)). This finding was validated on the GEO database (Figure 7(c)). Kaplan–Meier survival analysis of LGG patients suggests the reliability of MCL1 as a bad prognostic factor (HR = 1.66, 95% CI = 1.15–2.41, *P* < 0.01). MCL1 combined with four other markers to construct a new nomogram to predict the probability of survival at 1, 3, and 5 years of clinical diagnosis in patients with LGG based on patient’s WHO grade, gender, age and histological type (Figure 7(d)). Nomogram calibration curves validate the agreement between predicted and actual survival probabilities for LGG at 1, 3, and 5 years (Figure 7(e)).

### 3.7 Immune infiltration analysis of MCL1 in LGG

**Figure 8:**
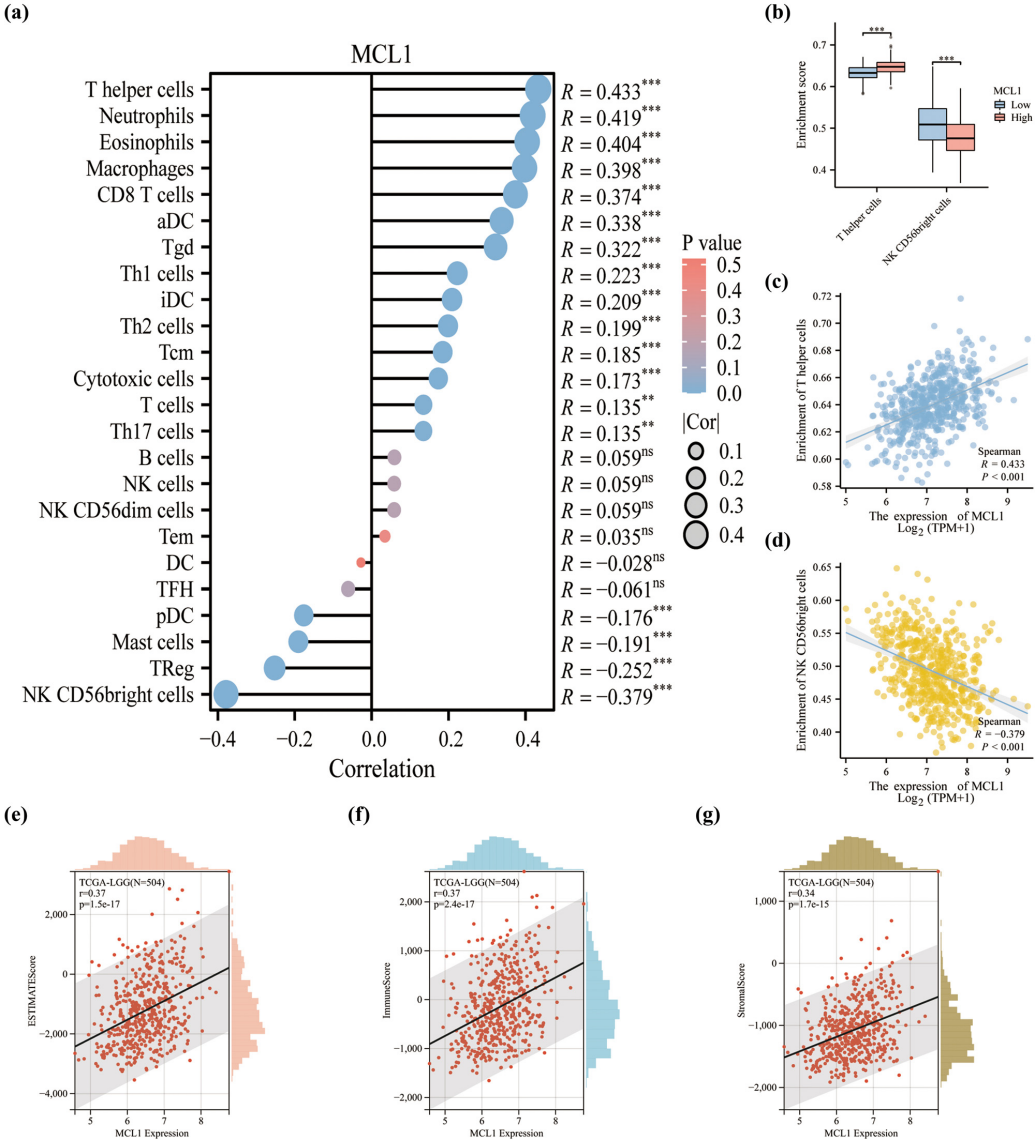
MLC1 expression is linked with immune infiltration in LGG based on TCGA. (a) Correlation between MLC1 and multiple immune cells. (b) MLC1 was associated with T helper cells and NK CD56bright cells. Correlation between enrichment scores and (c) MLC1 in T helper cells and (d) NK CD56bright cells. (e–g) Correlation between the expression level of MCL1 and three infiltration score. **p < 0.01, ***p < 0.001.

By performing the ssGSEA algorithm on 24 immune cells, we analyzed the results of the correlation between MCL1 and immune infiltration and presented them in the form of a lollipop plot (Figure 8(a)). Specifically, MCL1 was positively correlated with most immune cells such as T helper cells, neutrophils, and eosinophils and negatively correlated with NK CD56bright, TReg, and Mast cells. Among these immune cells, we specifically show a statistically significant correlation between T helper cells and NK CD56bright cells’ infiltration in the MCL1 differential expression analysis (Figure 8(b)). MCL1 expression levels were significantly positively and negatively correlated with the enrichment scores of T helper cells (Figure 8(c)) and NK CD56bright cells (Figure 8(d)), respectively. At the overall level of infiltration, we explore three immune infiltration scores that correlate with MCL1, including ESTIMATEScore (*r* = 0.37), ImnuneScore (*r* = 0.37), and StromaScore (*r* = 0.34). Results showed a meaningful positive correlation (all *p* < 0.001) (Figure 8(e)–(g)).

## 4 Discussion

The identification and characterization of cell death mechanisms not only promotes a fundamental understanding of cellular homeostasis but also provides important ideas for the treatment of many diseases such as cancer. Recent studies have identified a new form of disulfide-induced cell death in human cells, called disulfidptosis. This study suggests that GLUT inhibitor-induced disulfidptosis may be an effective strategy for treating tumors [12].

There has long been a search for better treatments for gliomas, particularly LGG, which has a relatively low stage and malignancy, can be stopped with aggressive treatment in some young people with the disease, and is the more promising of the glioma tumors to be cured [3,25]. However, traditional surgical resection combined with chemotherapy and radiotherapy is difficult to avoid tumor resistance and progression. Therefore, it becomes crucial to evaluate LGG prediagnosis and to investigate new drugs targeting specific functional pathways. Our study links disulfidptosis to the pathogenesis of LGG, identifies possible key genes through bioinformatics analysis, and explores potential therapeutic approaches.

In this study, we compared the expression of related genes in LGG tumors and normal tissues from the TCGA and GEO databases, and the data showed significant upregulation in tumor expression. We used GeneMANIA to predict functionally similar genes in hub genes to obtain 29 similar DRGs, most of which were positively correlated with each other. This defined gene set has scientific validity and reliability. The gene set KEGG enrichment analysis revealed enrichment of some apoptosis and disease-related pathways such as pathogenic *E. coli* infection, regulation of actin cytoskeleton, apoptosis-multiple species, ferroptosis, apoptosis, and so on.

This confirms the intrinsic pathway correlation between disulfidptosis and various cell deaths such as ferroptosis and apoptosis. Ferroptosis is a unique form of cell death that is driven by iron-dependent lipid peroxidation. Ferroptosis is closely associated with a variety of biological scenarios, including development, aging, immunity, and cancer [26]. In GO terms studies, we found that DRGs are involved not only in organelle outer membrane components and actin cytoskeleton organization but also in the VEGF receptor signaling pathway. The proven role of VEGF in promoting tumor angiogenesis and human cancer pathogenesis has led to the rational design and development of drugs that selectively target this pathway [27]. This partly explains the oncogenic role of disulfidptosis in LGG cells.

At the overall level, we perform a preliminary exploration of DRGs, which are strongly correlated with expression, immune infiltration, mutation, and drug sensitivity in LGG. Taking MCL1 for example, it ranked high in the list of methylation difference and was a significant prognostic risk factor for LGG.

MCL1 is a member of the BCL2 family and its high expression is closely associated with drug resistance in tumor [28]. MCL1 of expression also links to the pathway of Apoptosis, Cellcycle, DNA Damage, and other pathways, which is consistent with previous article studies [29]. For example, DNA damage induces apoptosis, which occurs in part through p53-responsive genes encoding pro-apoptotic BCL2 family proteins that bind to and inhibit anti-apoptotic Bcl-2 family members such as MCL1.

Based on consensus clustering, we identified two LGG subtypes (C1 and C2) by DRGs’ expression and found that the C2 subtype was associated with poor prognosis. On further analysis, C2 subgroup was more significant in relation to immune scores and immune checkpoints. The subgroups were then further explored for differential genes and finally found that enrichment analysis of the downregulated genes in the GO dataset is shown in T-cell activation, neutrophil activation involved in immune response, and neutrophil degranulation. This is further evidence of the importance of immunity and disulfidptosis in the development of LGG.

To identify new prognostic markers for LGG, we performed the LASSO regression analysis based on 29 DRGs for LGG patients in the TCGA database. We identified 14 genes with a disulfidptosis signature, including BAK1, SLC7A11, CYFIP1, WASF2, ABI2, BCL2L1, BID, TRIO, ABI1, SLC3A2, DPYSL2, MCL1, SLC25A25, and CYFIP2. We also confirmed that OS was significantly longer in the low-risk group than in the high-risk group. Meanwhile, the ROC time-dependent curves showed an accuracy of >0.70 for 1-, 3-, and 5-year survival rates. To verify the reliability of the above analysis based on TCGA data, further machine learning analysis was performed on LGG patients based on the GEO database. First, we used Limma analysis to identify a total of 1,533 upregulated genes and 1,818 downregulated genes, of which 7 were associated with DRGs, including MCL1, RAP1GDS1, WASF1, MFN2, CYFIP2, SLC3A2, and BOK. LASSO regression was then used to screen out the most relevant trait genes, MCL1 and RAP1GDS1. Meanwhile, we screened DRGs using RandomForest algorithm, showing the top 10 genes, including MCL1, RAP1GDS1, MFN2, SLC3A2, WASF1, CYFIP2, CYFIP1, DPYSL2, WASF2, and BOK. MCL1 was finally selected as the only candidate gene, a result that further demonstrates the role of MCL1 in LGG carcinogenesis. Previous studies have confirmed that MCL1 can promote cell migration and invasion in some types of cancers, including renal cell carcinoma [30], acute myeloid leukemia [31], and pancreatic ductal adenocarcinoma [32]. We further found that MCL1 expression was upregulated in LGG patients by analyzing data from TCGA and GTEx databases. This finding was validated on the GEO database. The reliability of MCL1 as a poor prognostic factor ultimately establishes nomogram as an aid to clinicians in the early clinical diagnosis of LGG.

Accelerated progression of tumors is not only associated with malignant cells but is also influenced by tumor microenvironment [33]. As researchers continue to learn more about the tumor microenvironment, there is great potential for further understanding of the relevant immune cell components and roles in the tumor microenvironment to guide immunotherapy [34]. We analyzed the results of the correlation between MCL1 and immune infiltration. MCL1 showed the most positive correlation with T helper cells and the most negative correlation with NK CD56 bright cells. T helper cells (CD4+ T cells) are essential for host defense but are also a major driver of immune-mediated disease [35]. For instance, multiple sclerosis is confirmed to be an autoimmune inflammatory disease caused by the recruitment of self-reactive lymphocytes (mainly CD4+ T cells) in the central nervous system [36,37]. Previous studies have revealed that immunity based on T helper cell characteristics of tumor subtypes affects prognosis and treatment response in breast cancer [38]. In addition, compared to the NK CD56 dim cells, NK CD56 bright cells are capable of producing large amounts of cytokines when monocytes are activated but have a lower natural cytotoxicity [39]. Therefore, studies focusing on one or more unique immune cells may help to identify potential mechanisms of action of MCL1 and demonstrate that MCL1 is a promising diagnostic LGG biomarker involved in immune regulation. Ultimately, MCL1-related studies and new targeted immunotherapies may help to improve the poor prognosis of patients and give physicians one more possibility to treat LGG.

Understanding the mechanisms and consequences of disulfidptosis may provide insights into novel therapeutic targets and strategies for cancer treatment. Currently, disulfidptosis has been shown to correlate with bladder cancer [40], hepatocellular carcinoma [12], and renal cell carcinoma [41], but an association with LGG has not yet been reported. MCL1 plays an important role in cancer development and has been associated with drug resistance in a variety of cancers. MCL1-selective inhibitors may represent a new class of anticancer agents that could provide clinical benefit to patients with a variety of hematological malignancies and solid tumors [42]. Furthermore, the literature suggests that exploring ubiquitination and deubiquitination of MCL1 is advancing therapeutic approaches and future directions [43]. This groundbreaking study provides novel insights and innovative findings by proposing, for the first time in the existing literature, a potential link between disulfidoptosis and LGG. The study further explores the significance of MCL1 expression of DRGs as a crucial prognostic factor in LGG.

This study has some limitations. We first explored DRGs using public databases such as GEO, TCGA, and GTEx but lacked clinical data of our own. This article lacks human tissue validation and further precise validation through biological experiments is needed. To address the heterogeneity of tumor samples, further techniques such as single-cell analysis or spatial transcriptomics allow for a more detailed characterization of the heterogeneity of cells within a tumor and their impact on gene expression patterns and immune infiltration

## 5 Conclusion

Our study reveals significant associations between DRGs and expression, immune response, mutations, and drug sensitivity in patients with LGG. We observed substantial heterogeneity among LGG patients, particularly within distinct disulfidptosis subclusters and DEGs. Importantly, we found that MCL1 may serve as a prognostic biomarker in LGG, predicting poor prognosis and correlating with levels of immune infiltration. These findings provide valuable insights into the potential use of MCL1 as a novel prognostic indicator and highlight its relevance for the development of new immunotherapeutic strategies. Our study represents a scientific and bold endeavor. Moving forward, further research should aim to explore and investigate the mechanistic aspects of MCL1’s role in LGG and explore potential therapeutic interventions based on these findings.
